# Infectious Trigger for Autoimmune Encephalitis: A Case Report and Literature Review

**DOI:** 10.1155/2019/5731969

**Published:** 2019-11-06

**Authors:** Najmus Sahar, Alexander Michael Nurre, Ryan Q. Simon

**Affiliations:** ^1^Wright State University Boonshoft School of Medicine, Miami Valley Hospital, Dayton, Ohio, USA; ^2^Boonshaft School of Medicine, Class 2019, Dayton, Ohio, USA

## Abstract

Herpes simplex virus 1 infection is a common cause of encephalitis (HSVE) in the United States. Post-HSVE development of N-methyl-D-aspartate receptor (NMDAR) antibodies resulting in autoimmune encephalitis is a rare complication, primarily affecting children and young adults. Anti-NMDAR develops 1–4 weeks after HSVE, manifesting as choreoathetosis and/or orofacial dyskinesia in children and psychiatric symptoms in young adults. We describe a case of a 61-year-old male who presented with agitation, behavioral changes, and confusion eight months after being treated for HSVE. Extensive investigation was unrevealing except for cerebrospinal fluid lymphocytic pleocytosis, a positive anti-NMDAR Ab titer 1 : 64, and imaging changes consistent with postviral encephalitis suggestive of HSV-induced anti-NMDAR encephalitis. Aggressive therapy resulted in limited success and persistent neurologic deficits. The unique features of this case are the old age of the patient and preceding HSVE which triggered this autoimmune process. Physicians should consider anti-NMDAR encephalitis in the differentials for relapsing patients after HSVE.

## 1. Introduction

Antibodies against N-methyl-D-aspartate receptors (NMDAR) have been discovered in patients following herpes simplex virus encephalitis (HSVE). This is believed to be associated specifically with the formation of antibodies against the GluN1 subunit of NMDAR, but the pathogenesis is not well understood [1]. Anti-NMDAR encephalitis is more commonly seen involving other etiologies such as ovarian teratoma, more common in younger women. The ovarian tumor in such cases produces heteromers related to the NMDA receptor [2]. The optimal treatment modalities of this disorder in older patients and the accompanying prognosis need further description. We now present a case involving an elderly male patient with the intent to communicate our experience and assist in the development of a treatment plan and improve the prognosis of future patients.

## 2. Case Report

A 61-year-old male with a history of coronary artery disease, hypertension, and hyperlipidemia presented to the hospital in April of 2017 with confusion and somnolence. On arrival, he was hemodynamically stable, and physical examination was significant only for delayed recall and poor concentration. Serum chemistries found hyponatremia, and cerebral edema was noted on the computed tomography (CT) scan of the head. A lumbar puncture revealed clear CSF fluid with 25 leukocytes/*μ*L (88% lymphocytes), 240 erythrocytes/*μ*L, and the protein level was 94 mg/dL (See Table 1). A Gram stain of the CSF revealed no organisms. The remainder of his tests, including serum chemistry panel, liver function panel, complete blood count, serum ammonia level, thyroid function test, and urinalysis, were normal. The urine drug screen and serum toxicology screen were negative. He was empirically started on intravenous (IV) vancomycin, cefepime, and acyclovir for presumed meningoencephalitis. Serum HIV and VDRL screens were negative. A magnetic resonance imaging (MRI) scan of the brain showed edema in the right frontotemporal lobes and left frontal lobe. Electroencephalograms (EEG) showed focal slowing in the right frontal/parietal regions without any epileptic discharges. The result of CSF HSV-1 polymerase chain reaction (PCR) test was positive, which confirmed the diagnosis of HSV-1 encephalitis. He was treated with 21 days of IV acyclovir and was eventually transferred to an inpatient rehabilitation unit. At the time of his discharge from rehabilitation, he continued to demonstrate severe cognitive and linguistic deficits. In June 2017, he was readmitted for pronounced confusion and erratic and aggressive behavior. A lumbar puncture was negative for HSV PCR, but a paraneoplastic CSF panel revealed anti-NMDAR antibodies suggestive of post-HSVE autoimmune encephalitis. He underwent therapy with serial plasma exchange (PLEX) cycles, but demonstrated mild improvement and remained far from his baseline personality and cognitive function. On discharge from the facility, his Montreal Cognitive Assessment (MoCA) score was 10/30 with significant deficits in the domains of visuospatial/executive, delayed recall, language, and orientation. The MRI of his brain at that time showed extensive signal intensities in the frontal and temporal lobes bilaterally.

**Figure 1:**
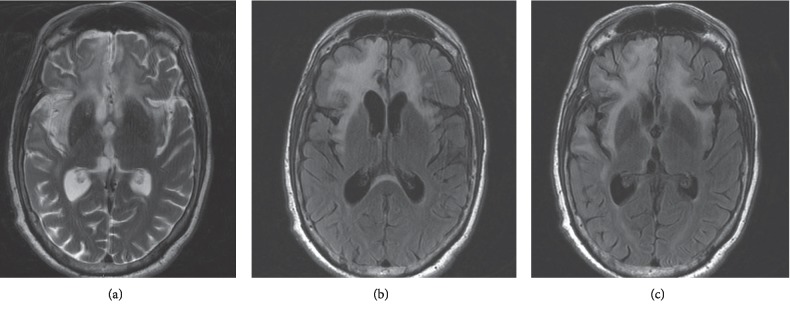
Brain MRI obtained during admission for anti-NMDAR encephalitis, 8 months after HSE onset. Increased T2 (a) and T2/FLAIR (b, c) signal (hyperintensity) in bilatéral frontaltemporal lobes (predominant on the right side).

**Figure 2:**
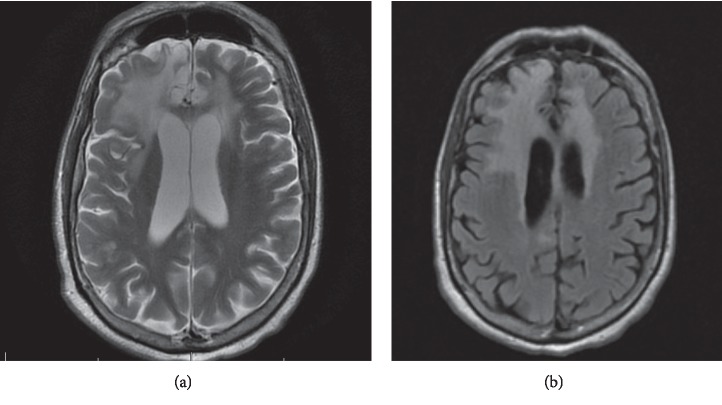
MRI brain after 11 months of HSVE. T2 (a) and T2/FLAIR (b) showed no additional changes other than the interval evolution of areas of encephalomalacia in right frontotemporal lobes.

In January 2018, he relapsed again with worsening agitation, confusion, and frequent mood swings. Brain MRI showed worsening enhancement of the previously affected areas (see Figure 1). CSF HSV PCR remained negative, and CSF studies did not suggest acute infection. CSF and serum anti-NMDAR antibody tests were positive with titers of 1 : 10 and 1 : 80, respectively (See Table 1). A positron emission tomography/CT scan did not show any occult malignancy, and expanded testing for infectious etiologies including toxoplasmosis and Lyme disease was negative. Serial PLEX therapy alleviated his symptoms reflected by improved MoCA scores ranging from 24 to 26/30 while inpatient. Repeat brain MRI in March 2018 did not show any new changes (see Figure 2). Outpatient therapy initiated with rituximab in April 2018 but in July 2018, had another acute decline treated with PLEX and steroid therapy. In September 2018, monthly cyclophosphamide therapy was started but four months into therapy, he suffered another relapse with worsening hallucinations. An MRI of his brain showed new faint cortical enhancement in the right anterolateral temporal lobe, and EEG showed seizures. He was managed with steroids and intravenous immunoglobulin (IVIG) therapy with optima control of his symptoms. He continued to suffer from intermittent episodes of combativeness, despite being on antipsychotic medications lacosamide and divalproex. He also experiences nighttime awakenings with confusion and has not achieved his baseline personality or cognitive function.

## 3. Discussion

HSVE-induced anti-NMDAR encephalitis is an autoimmune encephalitis more commonly seen in the pediatric population, making the treatment and identification of this disorder in older patients challenging. The California Encephalitis Project demonstrated that anti-NMDAR encephalitis is four times more common than viral etiologies of encephalitis in the cohort of patients under the age of 30, 65% of whom were below the age of 18 years [3]. Relapsing encephalitis caused by HSV is seen in a minority of patients, occurring in 7.1%–12.5% of adults and 14.3%–26.7% of children after an initial episode [4]. Discriminating anti-NMDAR encephalitis from recurrence of HSVE is important due to differences in treatment and prognosis. This can be done based on history, exam findings, HSV PCR, brain MRI findings, and response to antiviral treatment. Time to onset of symptoms of encephalitis is variable in HSVE but tends to occur 4–6 weeks following HSV infection due to anti-NMDAR antibody formation. New MRI lesions are uncommon with autoimmune disease in contrast to HSVE. Symptomology differs with HSVE and is characterized by focal neurological signs and abnormalities in behavior and movements. Autoimmune encephalitis will primarily manifest with abnormal behavior in adults [5]. Our patient had persistent behavioral issues after his initial episode of HSVE and was subsequently diagnosed with anti-NMDAR encephalitis two months after his initial diagnosis of HSVE.

The frequency of anti-NMDAR encephalitis in adults and elucidating risk factors that predispose a patient to acquiring autoimmune encephalitis are important questions for future research. One prospective observational study of 51 patients with HSVE examined the incidence of neuronal autoantibodies following the initial episode of encephalitis. Fourteen of their patients developed autoimmune encephalitis with all 14 developing some form of neuronal autoantibody (64% anti-NMDAR). However, 11 patients developed neuronal autoantibodies but did not develop any signs of clinical disease. A significant finding showed that the development of antibodies within three weeks of HSVE was a risk factor for the autoimmune disease [6].

Symptomology is different between pediatric and adult cases with adults experiencing more psychiatric symptoms and children experiencing a higher prevalence of neurologic symptoms [7]. Neurologic symptoms include seizures, dyskinesias (orofacial, choreoathetoid, and abnormal posturing), autonomic instability, and central hypoventilation [8]. Psychiatric symptoms include alterations in mental status, personality, psychosis, and catatonia [7]. In our patient, there was an overwhelming predominance of psychiatric manifestations and personality changes.

Our literature search on PubMed using key words “Anti-NMDAR encephalitis,” “post-HSV encephalitis,” and “autoimmune encephalitis” described case reports/series in adult patients with age over 18 [9–12]. These cases clearly demonstrate the prominence of psychiatric symptoms and absence of movement disorders. Only one case reported the presence of dyskinesia [9]. Common psychiatric symptoms included confusion, irritability, and suicidal ideation. The response to therapy and outcome was variable. Our patient had a persistent irritability and behavioral outbursts. Cases with more favorable outcomes had only minimal residual changes in personality, outbursts, aphasia, and memory. Different treatment options were utilized. One patient had almost complete resolution of symptoms with no treatment [10]. IV methylprednisolone was utilized in six of the ten patients and used alone in four patients [10–12]. In such a small group, we are unable to draw conclusions about the choice of treatment and the outcome.

First-line therapies are steroids, IVIG, and plasmapheresis alone or in combination. Second-line therapies include rituximab and cyclophosphamide. One study of 501 patients with anti-NMDAR encephalitis secondary to all etiologies (including teratoma) demonstrated a 53% improvement with the first-line therapy, 81% of which had a favorable outcome at 24 months. Of the 47% that did not improve, 57% of this group was given the second-line therapy of which 78% having a favorable outcome [7]. In a case report similar to our case, an elderly woman responded positively to IVIG, rituximab, and lastly a 3-month regiment of cyclophosphamide with only mild personality changes continuing after treatment [9]. Our patient demonstrated a strong improvement in his MoCA score some time after two rounds of first-line therapy with PLEX but has not shown significant improvement in his psychiatric symptoms and unfortunately, second-line therapy has demonstrated little efficacy.

## 4. Conclusion

We have described a case of HSVE-induced anti-NMDAR encephalitis in a 61-year-old male [13]. Physicians should consider anti-NMDAR encephalitis in the differential for relapsing patients after HSVE. Reemergence of neurologic and psychiatric symptoms should prompt analysis for HSV PCR and titers of antibodies against NMDAR [9]. Failure of the first-line therapy should be followed up with second-line treatment including rituximab with or without cyclophosphamide. This treatment regimen and long-term prognosis needs further evaluation and study particularly in adult population.

## Figures and Tables

**Table 1:** CSF analysis, HSV PCR, and anti-NMDAR Ab titer over time for our case.

CSF analysis	April 2017	June 2017	January 2018	March 2018	Ref. range
Appearance	Clear	Clear	Clear	Clear	Clear
WBC (% lymphocytes)	25 (95%)	21 (94%)	3 (95%)	4 (94%)	0–5 mm^3^
RBC	240	2	40	0	0–5 mm^3^
Protein	94	107	57	56	15–45 mg/dl
Glucose	ND	58	57	66	40–70 mg/dl
HSV PCR	Positive	Negative	Negative	Negative	Negative
Anti-NMDAR Ab	ND	Positive 1 : 64	Positive 1 : 10	Positive 1 : 20	Negative

CSF: cerebrospinal fluid, WBC: white blood cells, RBC: red blood cells, ND: not detected, HSV PCR: herpes simplex virus polymerase chain reaction, and anti-NMDAR Ab: antibodies against N-methyl-D-aspartate receptor.
